# A complete benchmark for polyp detection, segmentation and classification in colonoscopy images

**DOI:** 10.3389/fonc.2024.1417862

**Published:** 2024-09-24

**Authors:** Yael Tudela, Mireia Majó, Neil de la Fuente, Adrian Galdran, Adrian Krenzer, Frank Puppe, Amine Yamlahi, Thuy Nuong Tran, Bogdan J. Matuszewski, Kerr Fitzgerald, Cheng Bian, Junwen Pan, Shijle Liu, Gloria Fernández-Esparrach, Aymeric Histace, Jorge Bernal

**Affiliations:** ^1^ Computer Vision Center and Computer Science Department, Universitat Autònoma de Cerdanyola del Valles, Barcelona, Spain; ^2^ Department of Information and Communication Technologies, SymBioSys Research Group, BCNMedTech, Barcelona, Spain; ^3^ Artificial Intelligence and Knowledge Systems, Institute for Computer Science, Julius-Maximilians University of Würzburg, Würzburg, Germany; ^4^ Division of Intelligent Medical Systems, German Cancer Research Center (DKFZ), Heidelberg, Germany; ^5^ Computer Vision and Machine Learning (CVML) Research Group, University of Central Lancashir (UCLan), Preston, United Kingdom; ^6^ Hebei University of Technology, Baoding, China; ^7^ Tianjin University, Tianjin, China; ^8^ Digestive Endoscopy Unit, Hospital Clínic, Barcelona, Spain; ^9^ ETIS UMR 8051, École Nationale Supérieure de l'Électronique et de ses Applications (ENSEA), Centre national de la recherche scientifique (CNRS), CY Paris Cergy University, Cergy, France

**Keywords:** computer-aided diagnosis, medical imaging, polyp classification, polyp detection, polyp segmentation

## Abstract

**Introduction:**

Colorectal cancer (CRC) is one of the main causes of deaths worldwide. Early detection and diagnosis of its precursor lesion, the polyp, is key to reduce its mortality and to improve procedure efficiency. During the last two decades, several computational methods have been proposed to assist clinicians in detection, segmentation and classification tasks but the lack of a common public validation framework makes it difficult to determine which of them is ready to be deployed in the exploration room.

**Methods:**

This study presents a complete validation framework and we compare several methodologies for each of the polyp characterization tasks.

**Results:**

Results show that the majority of the approaches are able to provide good performance for the detection and segmentation task, but that there is room for improvement regarding polyp classification.

**Discussion:**

While studied show promising results in the assistance of polyp detection and segmentation tasks, further research should be done in classification task to obtain reliable results to assist the clinicians during the procedure. The presented framework provides a standarized method for evaluating and comparing different approaches, which could facilitate the identification of clinically prepared assisting methods.

## 1 Introduction

Colorectal cancer (CRC) is the third most common cancer in both genders and the second leading cause of death in the world. Globally, 1.9 million new cases of CRC are diagnosed annually, with an incidence rate slightly higher in men (1). Almost all CRCs originate from polyps, which are abnormal tissue growths that appear along the colon.

Colonoscopy is the gold standard tool for CRC detection, since it allows *in-situ* polyp identification and extraction. Once the polyp is detected, common protocols indicate that it should be removed to perform a posterior histological analysis to determine the degree of malignancy of the lesion and, therefore, prescribe a treatment to the patient. Resecting all polyps in the colon increases the total exploration time and, due to the resection process itself, the risk of colon perforation is also increased.

From a histological point of view, polyps can be classified as adenomatous and non-adenomatous, depending on whether or not they present a risk of degeneration. Under this classification, non-adenomatous lesions include hyperplastic and sessile polyps whilst the adenomatous class include remaining polyp types which could transform to malignant tumor. Taking all this into account, resecting non-adenomatous polyps could represent a waste of resources and exposing the patient to an unnecessary surgical procedure. Also, final diagnosis has an unavoidable time gap because the analysis of the biopsied tissue has to be done afterwards.

In recent years, several classification standards have been designed to improve histological prediction in the exploration room. Most of them rely on image magnification and lens pigmentation, aiming at improving the quality of visualization of patterns over the polyp tissue. Unfortunately, the use of some of these systems, particularly those using virtual chromoendoscopy, can result in a dependence of a specific manufacturer, limiting their widespread use.

Strategies like *resect and discard* or *leave in situ* can only be used by expert endoscopists with a good adenoma detection rate (ADR) as colonoscopy heavily relies on non-quantifiable visual assessment of polyps. According to (2, 3), the overall adenoma miss rate is still around 26%. This can be critical to the patient if he/she does not undergo for a new exploration in the following years, as survival rate greatly depends on the stage the lesion is detected on (4).

Considering all this, there is a need and an opportunity of computer-aided support systems that can help clinicians in two tasks: detecting the lesions during the colonoscopy exploration and, once they are detected, to assess their malignancy degree in order to guide clinicians in the decision regarding whether the lesion should be removed or not. Regarding the latter, having such a system would facilitate the transition to the *resect and discard* protocol, which proposes to remove only the potentially malignant polyps while leaving *in situ* the rest. This standard reduces both the exploration time and the perforation risk.

We foresee this computer-aided support system as a complete pipeline where the lesion is first detected and then segmented to allow the system to focus on the analysis of the texture pattern of the lesion to determine its final histology. Taking this into account, we can divide computational methods for polyp image into three tasks: detection, segmentation and classification.

Regarding detection, a given computational method should be able to correctly determine polyp presence/absence in a set of short colonoscopy sequences all showing a polyp in some of the image frames: in case a polyp is present, the system should be able to display the detection output above the polyp.

With respect to polyp segmentation, the objective is that the output of the method matches the ground truth mask. Considering that segmentation is meant to be used as part of a classification pipeline, we validate this task in both standard definition and high definition still images, where more texture within the polyp can be observed.

Finally, we explore the potential of intelligent systems to deal with polyp classification in high definition images, where differences in texture patterns within the polyp can be key to differentiate between benign and malign lesions.

During the last two decades, several efforts have been made to develop and validate computer-aided support systems for colonoscopy, being the majority of them focused on the polyp detection task. Unfortunately, they are commonly tested on private datasets which raises questions about the validity of the results presented in the different contributions. Moreover, when presenting the results the vast majority of them ignore aspects crucial to a potential deployment in the exploration room such as processing time or reaction time.

We present in this paper a complete validation framework to assess the performance of polyp detection, segmentation and classification methods. This includes both the definition of datasets and evaluation metrics as well as proposing different validation experiments that go beyond the analysis of individual performance of a given method. As a proof of concept of the proposed validation framework, we present for the first time a complete comparison analysis in the scope of a recent MICCAI challenge.

The main contributions of this paper are:

- Definition of a common framework for validation of multiple tasks (detection, segmentation and classification) related to colonoscopy images.
- Introduction of CVC-HDClassif dataset: a completely labelled public dataset for polyp classification.
- Presentation of the results of a comparative study of several methodologies presented in recent MICCAI challenges.

The rest of this paper is structured as follows: In section 2 we describe the related work for polyp localization, segmentation and classification. In section 3 we present the complete validation framework, including the introduction of novel CVC-HDClassif dataset. In section 4 we present the methodologies that will be part of the comparison study. In section 5 we show results of this comparison study. Finally, in section 6 we present some of the main findings after analyzing the performance of the different methods. We close this paper with the main conclusions and future work.

## 2 State of the art

In this section, we review recent computational methods that tackle the different stages of polyp characterization, specifically focusing on detection, segmentation and classification tasks. It has to be noted that methods presented in this section encompass works that deal only with traditional colonoscopy images, excluding from this review those works that use Wireless Capsule Endoscopy (WCE) images.

### 2.1 Polyp detection and localization

Polyp detection and localization methods can be broadly categorized into real-time and non-real-time approaches; real-time methods typically leverage YOLO networks or their derivatives. For instance, Zhang et al. (5) integrated a module to re-score confidence using Efficient Convolution Operators (ECO) to track detected polyps, thereby reducing false positive rate without compromising the real-time performance. Similarly, Yang et al. (6) introduced YOLO-OB, a model addressing the challenges related to polyp size variability. Their approach integrates a bidirectional multiscale feature fusion structure which, combined with an anchor-free box regression strategy, demonstrated significant improvements in detection of small polyps.

Non-real-time methods often exploit temporal dependencies or use heavy ensembles to improve detection accuracy. Qadir et al. (7) used Faster R-CNN and aimed at improving precision and specificity by introducing a false positive reduction module that exploits temporal dependencies between consecutive frames, effectively reducing false positives without compromising sensitivity.

Kang et al. (8) used an ensemble of detectors with different feature extractors, later post-processing the outputs to refine bounding boxes and instance masks by learning how to weight each prediction. Zheng et al. (9) approached detection as a tracking problem using optical flow, supplemented by a fine-tuned box regressor to handle tracking failures on the fly. Ma et al. (10) utilized bootstrapping for test-time adaptation in video sequences, applying temporal consistency techniques to refine predictions. Lastly, Jia et al. (11) extended Faster R-CNN with a polyp proposal stage, also providing segmentation masks for the localized polyps.

### 2.2 Polyp segmentation

Research during recent years in medical image segmentation has been dominated predominantly by U-net (12) like models, which consists of an encoder-decoder network with skip connections that enables to capture effectively both global and local context. Available literature presents more sophisticated models like Unet++ (13), which consisted of using multiple U-Nets with varying depths and densely connected decoders at the same resolution by using skip pathways to address the optimal depth problem. Another example of encoder-decoder network is DUCK-Net (14), which presents a model capable of effectively learning from small amounts of medical images and generalizes well. This model uses an encoder-decoder structure with a residual downsampling mechanism and a well-tailored convolutional block to capture and process image information at multiple resolutions in the encoder segment.

Attention mechanisms have further enhanced the performance of segmentation models by allowing networks to focus on relevant parts of the image. For instance, Pranet (15) proposed a parallel reverse attention in order to address first diversity of size, color and texture from the polyps and, second, the irregular boundary problem generated by the surrounding mucosa. Their methodology aggregated high-level features in order to generate a guidance area and used reverse attention to generate boundary cues.

In recent years, transformer-based architectures have motivated a shift in medical image segmentation due to their capacity of capturing long-range dependencies. Dong et al. (16) introduced the use of Pyramid Transformers as the encoder, including three different modules to handle specific polyp properties: 1) a cascaded fusion module (CFM) to collect semantic information from high-level features; 2) the camouflage identification module (CIM) which focused on low-level features and 3) the similarity aggregation module (SAM) that fused cross-level features.

In Polyp2Seg (17) the authors adopted a transformer architecture as its encoder to extract multi-hierarchical features. The authors of this work added a novel Feature Aggregation Module (FAM) to progressively merge the multi-level features from the encoder to better localize polyps by adding semantic information. Next, a Multi-Context Attention Module (MCAM) removed noise and other artifacts, while incorporating a multiscale attention mechanism to improve polyp detections.

Finally, B. J. Matuszewski et al. proposed two transformer-based architectures; the first one being a full-size segmentation model named Fully Convolutional Branch Transformer (FCN-Transformer) (18) and the second one being a new CNN-TN hybrid model named FCB-SwinV2 Transformer (19).

### 2.3 Polyp classification

In recent years, polyp classification methods have progressed from traditional approaches towards the use of convolutional neural networks and, lately, to transformer-based architectures. Examples of such traditional methods can be found in the study by Sanchez-Montes et al. (20), where the authors present a method to classify polyps into dysplastic and non-dysplastic lesions by extracting a set of hand-crafted features based on contrast, tubularity and branching level of the region. Lesions were then classified by using a set of SVM and a voting system.

Byrne et al. (21) method, which worked under real-time constraints. differentiated between adenomatous and non-adenomatous polyps using Narrow Band Images (NBI). Their method used a recurrent system to re-score predictions confidence by taking into account previous predictions, assuming that the images come from the same sequence.

Following this, the advent of CNNs supposed a clear revolution in the field of polyp classification, as shown by the work of Lui et al. (22) where the authors present a method that aims to distinguish treatable lesions from non-reversible ones by using a convolutional network. Their method worked well with NBI and WL images, but they noticed that the features extracted from NBI images provide better predictions. In the work of Patel et al. (23), the authors provided a benchmark on multiple datasets (4 different polyp datasets concatenated) that contained two different histological classes. They concluded that sequence-base performance is less consistent than frame-based due to the significant appearance changes along the sequence.

The shift towards transformer models is exemplified by several works. For instance, Krenzer et al. (24) presented a tranformmer network whereas texture information is analyzed following NICE paradigm using a few-shot learning algorithm based on the Deep Metric Learning approach, enabling an accurate classification even in those cases where data is scarce.

In Swin-Expand (25) the authors proposed a fine-grained polyp segmentation method that incorporates a simple and lightweight decoder and a modified FPN to enrich features into the existing Swin-Transformer architecture. Finally, in PolypDSS (26) the authors presented a computer-aided decision support system that integrates locally shared features and ensemble learning majority voting strategies to assist clinicians in both polyp segmentation and classification tasks.

## 3 Validation framework

In this section we present the complete validation framework that we propose for the assessment of the performance of polyp characterization methods. Taking this into account, we introduce the several datasets that will be used in the different validation experiments, we explain the annotations they contain and how they have been generated, as well as we present the metrics that will be used to represent method’s performance.

### 3.1 Datasets

In biomedical domains it is usually difficult to find datasets with large amount of annotated, high quality and varied samples, contrary to what happens with general purpose datasets like ImageNet (27), OpenImages (28) or MSCOCO (29). This is due to limited access to the primary data, high costs related to acquisition and the excessive time often needed for annotation.

Biomedical datasets require to be annotated by experts in the field to assure the high quality of the annotations. With respect to colonoscopy image analysis, most of the studies use a combination of public and private datasets, making it difficult to establish a fair comparison between methods.

Nevertheless, there already exist a wide variety of colonoscopy image and video datasets, as it can be seen in **Table 1**. As it can be seen, the majority of them have been designed to assess the performance of polyp detection methods although only a few of them (CVC-ClinicVideoDB, ASU-Mayo Clinic Colonoscopy Video, Colonoscopic Dataset, PIBAdb and PolypGen) include fully annotated video data. With respect to polyp classification, it is interesting to mention that no available dataset goes beyond two different classes in the data cohort, dividing existing data into benign and malign polyps without paying particular attention to clinically relevant categories such as serrated sessile adenomas.

**Table 1:** Comparison of public datasets for polyp detection, segmentation and classification.

Dataset	Format	Image type	Resolution (w x h)	Ground Truth	Images	Sequences	Patients	Task
CVC-ClinicDB (30)	Image	WL	384 × 288	Binary masks	612	31	23	Detection Segmentation
CVC-ColonDB (31)	Image	WL	574 × 500	Binary masks	300	13	13	Detection Segmentation
CVC-EndoSceneStill (32)	Image	WL	574 × 500 384 × 288	Binary masks	912	N/A	N/A	Detection Segmentation
CVC-PolypHD (33)	Image	WL	1920 × 1080	Binary masks	56	N/A	N/A	Detection Segmentation
ETIS-Larib (34)	Image	WL	1225 × 966	Binary masks	196	34	N/A	Detection Segmentation
Kvasir-SEG (35)	Image	N/A	Multiple resolutions	Binary masks Bounding boxes	1000	N/A	N/A	Detection Segmentation
CVC-ClinicVideoDB (36)	Video	WL	768 × 576	Binary masks	28563	38	N/A	Detection Segmentation
ASU-Mayo Clinic Colonoscopy Video (37)	Video	WL/NBI	688 × 550	Binary masks	N/A	38	N/A	Detection
Colonoscopic Dataset (38)	Video	WL/NBI	768 × 576	Polyp classification	N/A	76	N/A	Classification
PICCOLO (39)	Image	WL/NBI	854 × 480 1920 × 1080	Bounding boxes Polyp classification	3433	N/A	40	Detection Segmentation Classification
LDPolypVideo (40)	Video	N/A	768 x 576 (videos) 560 × 480 (images)	Bounding boxes	40187	160	200	Detection Segmentation
KUMC dataset (41)	Image	WL/NBI	Multiple resolutions	Bounding boxes Polyp classification	37899	80	N/A	Detection Segmentation Classification
CP-CHILD-A, CP-CHILD-B (42)	Image	N/A	256 x 256	Positive vs negative frames	A:8000 B:1500	N/A	N/A	Detection
SUN (43)	Image	N/A	1240 x 1080	Bounding boxes	49136	N/A	100	Detection
Colorectal Polyp Image Cohort (PIBAdb) (44)	Video/Image	WL/NBI	768 × 576	Bounding boxes Polyp classification	boxes	N/A	1176	Detection Classification
POLAR (45)	Image	NBI	N/A	Bounding boxes Polyp classification	2637	N/A	1339	Detection Classification
NBIPolyp-UCdb (46)	Image	NBI	576 × 720	Binary masks	86	11	N/A	Detection Segmentation
WLPolyp-UCdb (47)	Image	N/A	576 × 720	Not disclosed	1680	42	N/A	Detection
PolypGen (48)	Video/Image	N/A	N/A	Binary masks	1537	N/A	N/A	Detection
BKAI-IGH NeoPolyp-Small (49)	Image	WLI/FICE	N/A	Binary masks	1200	N/A	N/A	Segmentation
Gastro-Vision (50)	Image	WLI/NBI	Multiple resolutions	Anatomical landmarks Pathological abnormalities Polyps findings	8,000	N/A	N/A	Classification

N/A: Not provided.

In this subsection we focus on the datasets that we included in the validation framework; as well as presenting the details of each of them, we explain the acquisition and annotation procedures. All the datasets used in this paper will be fully disclosed and made publicly available upon paper publication in the following address https://pages.cvc.uab.es/ai4polypnet/datasets.

#### 3.1.1 Polyp detection

CVC-VideoClinicDB dataset, originally published in (36), is composed by 36 video sequences, each of them containing at least one polyp and acquired from a different patient. The video sequences show different colon explorations with white light endoscope and they were obtained using Olympus EndoBase software at Hospital Clinic of Barcelona, Spain. Endobase provides a video output with 384×288 resolution, sequences being recorded at 25 fps.

The 36 sequences were divided into training (15 sequences, 9830 images), validation (3 sequences, 2124 images) and test (18 sequences, 18733 images) subsets. **Table 2** shows the number of positive (PF) and negative (NF) frames per video.

**Table 2:** Content of the CVC-VideoClinicDB dataset. In the first column, videos 1 to 15 refer to the training split, whilst 16 to 18 refer to the validation.

Training and validation dataset			Test dataset		
Video	PF	NF	Video	PF	NF
1	386	112	1	365	1351
2	597	176	2	302	0
3	819	153	3	638	52
4	350	40	4	921	99
5	412	78	5	1354	1256
6	522	335	6	454	0
7	338	103	7	1116	283
8	405	44	8	773	187
9	532	19	9	632	136
10	762	78	10	191	0
11	370	130	11	1185	0
12	261	124	12	270	240
13	620	4	13	327	0
14	2015	45	14	778	349
15	360	215	15	1103	71
16	366	5	16	767	817
17	651	146	17	1165	765
18	259	122	18	251	538

**Figure 1:**
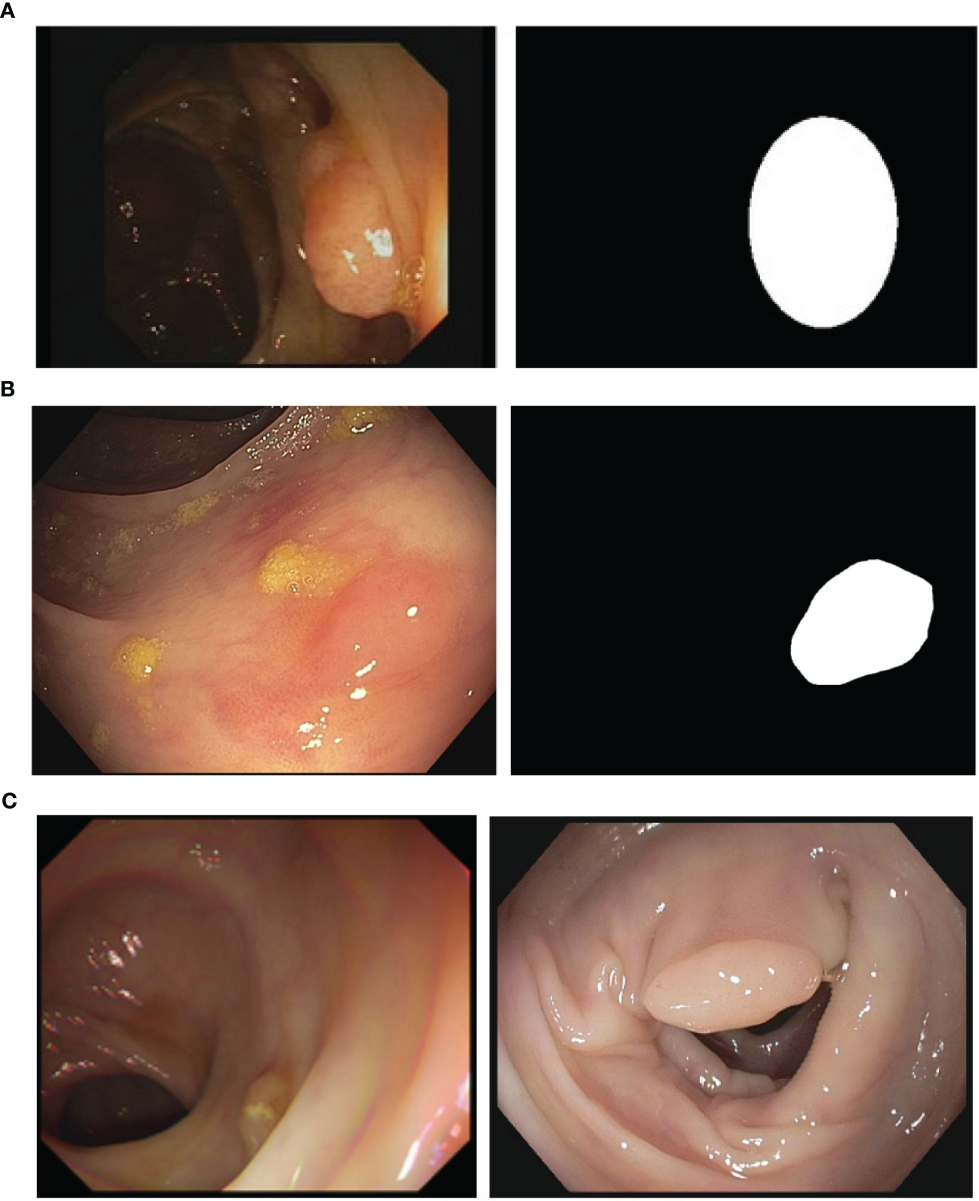
**(A)** A sample image and corresponding annotation mask from the CVC-VideoClinicDB (36). **(B)** Sample image and the corresponding annotation mask from the CVC-PolypHD. **(C)** Sample images from CVC-HDClassif labeled as adenoma (left) and non-adenoma (right).

With respect to the annotations, clinicians provided for each image as ground truth a binary mask in which each of the polyps present in the image is approximated as an ellipse. GTCreator (51) was used as annotation platform as it allowed clinicians to easily transfer annotations within consecutive frames, speeding up the ground truth generation process. We show in **Figure 1A** an example of a frame extracted from one of the sequences of CVC-VideoClinicDB alongside its ground truth.

#### 3.1.2 Polyp segmentation

Regarding polyp segmentation, two different sets are provided: standard definition (SD) and high definition (HD). SD dataset contains a total of 912 images distributed in training and test set with 300 (CVC-ColonDB, originally published in (31), extracted at Beaumont Hospital and St. Vincent’s Hospital in Dublin, Ireland) and 612 images (CVC-ClinicDB, originally published in (30), extracted at Hospital Clinic of Barcelona, Spain) respectively. The images from the training set have a resolution of 574×500 whilst the test set images have a resolution of 384×288.

Images from both sets were individually extracted by clinicians during the observation of several colonoscopy sequences (13 for the case of CVC-ColonDB and 31 for CVC-ClinicDB). Special attention was kept to ensure similar views from a given polyp were not included in the final dataset. With respect to the ground truth, it consisted of binary masks covering exactly all pixels belonging to the polyp in a given image. Annotations were created using Adobe Photoshop.

With respect to HD data, we use CVC-PolypHD dataset, which contains a total of 164 images, all extracted using an external frame grabber connected to Olympus Exera processing tower. We provide as annotation a pixel-wise binary mask covering the polyp in the image, made by clinicians at Hospital Clinic using GTCreator software. We show in **Figure 1B** an example of image data and corresponding ground truth. Images from this dataset were acquired with the authorization of the Clinical Research Ethics Committee (CREC) of the Hospital Clínic de Barcelona (HCB) with reference HCB/2014/1148.

#### 3.1.3 Polyp classification

CVC-HDClassif dataset is composed by a total of 1126 still high definition images, each of them containing a single polyp. There are a total of 471 unique polyps, with a variable number of shots per polyp (between 1 and 23). Special attention was paid to ensure that images from the same polyp showed a completely different view of the lesion.

These images were obtained using an external frame-grabber that captures the output signal from a white light endoscope and produces uncompressed images with HD resolutions: (1920×1080) or (1350×1080) depending on the used endoscope. Images from this dataset were acquired with the authorization of the Clinical Research Ethics Committee (CREC) of the Hospital Clínic de Barcelona (HCB) with reference HCB/2014/1148.

CVC-HDClassif dataset presents 3 stratified splits; train, validation and test with 788/113/225 images and 329/49/93 unique polyps respectively. **Table 3** describes the dataset, which presents an imbalance between the two classes (Adenoma: 69.7%; Non-adenoma: 30.3%). Images from the same polyp were always kept in the same split.

**Table 3:** Clinical metadata associated to the different polyps in CVC-HDClassif dataset.

	Train	Val	Test	All
Classes				
**Adenoma**	526	77	147	750
**Non-adenoma**	262	36	78	376
Localization				
**Rectum/Sigma**	339	65	132	536
**Other**	449	48	93	590
Size				
***<*=5 mm**	327	48	87	462
**6 − 10**	153	29	39	221
***>*=10 mm**	308	36	99	443

With respect to the localization distribution, 44% of the images are from polyps located on rectum and sigma. Out of all the polyps acquired, 43% of the total polyps are considered diminutive (less than 5mm) and 19% are small (between 6 and 10 mm): these are the ones that are more difficult to detect and classify by expert clinicians.

Each image contains only one instance for which we provide the histological class, where we can differentiate between non-adenomatous (NAD) and adenomatous (AD) polyps and a pixel-wise segmentation with the polyp region.

The annotations for this dataset were completely generated with the help of GT-Creator annotation tool (51). For each image that contains one or more polyps, we provide three different annotations: a binary mask that contains the regions with polyps, a set of bounding boxes containing the minimum bounding box for each of the binary mask regions and a set of class IDs that match each of the bounding boxes. Segmentation masks were done by multiple expert clinicians. Bounding boxes are automatically computed from the binary mask.

The class id corresponds to the actual histological class of the lesion obtained after pathology analysis of the lesion. We distinguish two different classes, corresponding to nonadenomatous (NAD) and adenomatous polyps (AD). In **Figure 1C** we provide a sample from the polyp classification dataset along with its corresponding ground truth binary mask.

### 3.2 Metrics

We propose to measure polyp detection performance by using Mean Average Precision (mAP), which is the gold standard metric to evaluate methods performance. Average Precision (AP) can be defined as the area under the precision-recall curve; considering this, mAP is defined as the average of the AP over all classes and gives the overall performance of the method. Besides providing overall mAP score, we also provide results for two specific points in the curve, *mAP*_50_ and *mAP*_75_ which aim to represent, respectively, acceptable and good detections. Apart from mAP, we also provide common metrics such as Precision, Recall, Specificity, Accuracy, F1 and F2-scores.

Besides, we also provide Reaction Time (RT) for each of the methodologies compared, aiming to measure how fast a given polyp detection method reacts to polyp presence in the endoluminal scene. We define RT as the difference (in number of frames) between the first appearance of a polyp in a video sequence and the first correct detection provided by a given method. In this context, we label a detection as correct if it has at least a 0.5 of Intersection over Union (IoU) with respect to the ground truth.

Regarding polyp segmentation, we use common Intersection over Union and DICE scores. With respect to polyp classification, we base the analysis of the performance by means of the calculation of the confusion matrix, which includes the use of common performance metrics such as Precision, Recall, Specificity, Accuracy, F1 and F2 scores as well as Matthew’s Correlation Coefficient (MCC). Also, due to the clinical nature of the task we are also taking into account valuable metrics for the clinicians, such as Negative Predictive Value (NPV), which focuses on how a given method is able to correctly categorize the positive class (non-malignant lesion in this context), which is one of the indicators used to determine the feasibility of the use of CAD systems in the application of protocols such as resect and discard.

## 4 Methodologies

We present in this section the key details of the methodologies used by each of the teams that took part on the GIANA 2021 challenge. **Table 4** shows a summary of the different methodologies.

**Table 4:** Summary of information from the teams that took part in any sub-challenge from GIANA 2021.

Team	Base architecture	Changes over architecture	Implementation details	Hardware
AI-JMU	YOLO V5	Added REPP to reduce false positives Gradual finetuning	Standard practices	1x RTX 3080 (45 FPS)
AURORA	DETR-ResNet50	Base architecture	Intensive DA	Not provided
BYDLab	Faster R-CNN (FPN-ResNext-101)	Multi-scale training OHEM	Intensive DA	Not provided No real time
CVC	Faster R-CNN (Swin-Transformer Tiny)	Base architecture	AutoAugment Test-Time Augments	1x RTX-2070 (30 PS)
a) Detection challenge				
Team	Base architecture	Changes over architecture	Implementation details	Hardware
AURORA	MiT-B5	Losses: focal; Dice	Keep the largest region over certain threshold	Not provided
CVC	SegFormer-B0	Losses: CE; Dice	TrivialAugment policy	1x RTX-2070 (50 FPS)
HK-UST	Unet	Base architecture	Standard DA	Not provided
UoN	DeepLab V3	GRU layer replaces ASPP	Standard DA	Not provided
UPF	Double Encoder-Decoder (FPN-ResNext-101)	Losses: Dice Sharpness-Aware Minimization	Merged SD and HD	Not provided Not real time
b) Segmentation challenge				
Team	Base architecture	Changes over architecture	Implementation details	Hardware
AURORA	MiT	Coarse and fine heads for classification and fine-grained, respectively	Intensive DA Test-Time augmentations	Not provided
BYDLab	Faster R-CNN	Multi-scale training OHEM	Keep top-1 as prediction	Not provided No real time
CVML	EfficientNet-V2	Base architecture	Crop the endoscope mask 5-fold ensemble	1x RTX 3090 (25 FPS)
Team AB	EfficientNet B7	Knowledge distillation	Three steps: 1. Train teacher 2. Distill the model 3. Fine-tune student with segmentation as proxy task	1x RTX-3090
UPF	Double Encoder-Decoder (FPN-ResNext-101)	Same as segmentation Losses: 3-class CE loss; BCE (adenoma); BCE (polyp)	AutoAugment Test-Time Augments	Not provided Not real time
c) Classification challenge				

If not specified, assume the authors follow the standard implementation. DA stands for data augmentation; CE loss refers to cross-entropy loss.

### 4.1 AI-JMU

This team used YoloV5 (52) as their base architecture for polyp detection, since real-time is a well-known architecture due to its proficiency in real-time object detection. The authors modified the original work by adding real-time robust and efficient post-processing (REPP) (53) in order to reduce the false positive rate and increase the consistency over consecutive frames.

The proposed training pipeline consists of two steps: They start with pretrained weights on MS-COCO Dataset and fine-tuned to the challenge data. The training is performed by doing progressive fine-tuning: starting with the last two layers and the REPP block and progressively adding layers until the whole net is being trained. After this, they keep training the whole network until the model stops improving in the validation set results.

### 4.2 AURORA

With respect to the detection task, they used DETR (54) architecture with ResNet-50 as backbone. The model was pretrained on COCO and fine-tuned on the challenge data. They used the following transformation for augmenting the images during training: Random Brightness, ColorJitter, GaussianBlur, RandomFlip, RandomResizedCrop and Random Sharpness.

For the segmentation task, they used Mix Transformer (MiT) architecture (55), concretely the B5 model. The model was trained using focal loss and dice loss. They also post-processed the output in order to only keep the most confident region of each image.

For classification task they took the MiT encoder as the backbone. Then the methodology passed the encoded features through a neck module to merge the multi-level features. Finally, they used two parallel heads in order to predict coarse-grained and fine-grained predictions. The coarse one predicted the final class, whilst the fine-grained kept the spatial information to perform dense predictions. Images are resized to 512×512 and data augmentation was applied during training and testing time.

### 4.3 BYDLab

For polyp detection they used Faster R-CNN (56) architecture with FPN-ResNext-101 as backbone. The use of FPN in this architecture allows to effectively combine low-resolution features with high-resolution features in order to obtain stronger semantical features. The model was pretrained on COCO and fine-tuned on the challenge data. Regarding the data, they trained using multiscale images and applied the standard image augmentation protocol (random crop, rotations) and additionally, brightness, contrast and saturation augmentations. Finally, to mitigate the false positive, they added to their training Online Hard Negative Example Mining (OHEM) (57) to keep the positive negative ratio for each batch around 1:3. While for the detection task the authors kept all the predictions over a certain score threshold (0.5), they used only the class associated to the most confident prediction as the output for the image classification task.

### 4.4 CVC

For the detection task they used Faster R-CNN with Swin-Transformer as backbone. They fine-tuned a model that was previously trained on COCO object detection task. Their methodology used Autoaugment (58) to learn to resize and crop policies as well as standard data augmentation transforms (flips, ColorJitter, and blur). They also used Multiscale Test augmentation for generating the predictions.

For segmentation tasks they relied on Segformer (55), which was trained minimizing the cross entropy plus dice loss. They used TrivialAugment (59) policy for the data augmentation and resizing the images to a 512×512 resolution. The predicted masks were resized back to their original size.

### 4.5 CVML

To solve the classification challenge the CVML team used an ImageNet pre-trained EfficientNetV2 architecture (60) fine-tuned on the GIANA data using 480×480 image resolution. The adopted solution did not use ground truth segmentation data in the design of the classifier. The EfficientNetV2 architecture was selected after a performance comparison between other popular image classification architectures (including the Vision Transformer and EfficientNetV1 architectures). They decided to go with EfficientNetv2 architecture instead of Vision Transformers as preliminary studies on the latter shown that they did not achieve good results on small datasets, as transformer architectures are more data-hungry than convolutional neural networks.

Data is pre-processed by removing image background (endoscope generated mask). For data augmentation during training, standard transformations (flips, transposes, and rotations) and image warping (via the use of thin plate splines to varying random degrees) is used. The network design parameters were selected based on 5-fold cross validation experiments using the training data.

### 4.6 HK-UST

For the segmentation task, they fused both SD and HD datasets, and trained a UNet-based model following standard practices. They go with U-Net since it is a well-established model for segmentation that is an encoder-decoder model where the encoder learns to capture correctly the context and the decoder learns to combine and reconstruct the lower resolution with the skip connections from the encoder. Those skip connection enable to recover the details that would be lost along the encoder path and enables a fine-grained delineation of segmentation masks.

### 4.7 Team AB

Their classification method takes advantage of the provided segmentation annotations to guide the model towards learning additional spatial information that is relevant to classify correctly the polyps. For this they rely on EfficientNet-B7 (61) as their architecture and define a 3-steps pipeline for training their model. First they pre-train the model for the classification task; then they perform knowledge distillation using the previous model as teacher and finally a fine-tune step is performed over the distilled model where the segmentation and the classification are optimized together.

### 4.8 UoN

For segmentation challenge they relied on DeepLab-V3 (62) but modifying the ASPP block. Concretely, they changed the adaptive image pooling by a Gated Recurrent Unit (GRU) (63) in order to capture the contextual information within the feature maps in order to enhance the segmentation capabilities of DeepLab architecture. This simple change is motivated by the fact that GRU is capable of modelling long-range dependencies within an image by the feature map as a sequence, whilst adaptive average pooling has no learnable parameters and cannot capture those long-range dependencies effectively.

### 4.9 UPF

For the segmentation task they modified Double Encoder-Decoder Networks (64) but they differentiate from this work in three key aspects: first they use ResNext101 as encoder instead of the one in the original work to increase the learning capabilities, and they used a FPN (65) as decoder with the purpose of increasing the receptive field; second, they performed optimizations by Adam with Sharp- Aware Minimization (SAM) (66) and finally, they merged both SD and HD datasets into one with common resolution of 512×512. They trained the model by early-stopping when Dice score stops improving on each separate validation set.

For the classification task they took their segmentation approach and trained it with extra losses to minimize: a) 3-class Cross-Entropy (background, adenomatous, non-adenomatous), b) Binary CE computed by accumulating both positive class (background, rest) and c) Binary CE for the probability of being adenomatous (defined by the probability of being adenomatous over the sum of probabilities of both classes).

## 5 Results

In this section we present the summary of the results achieved for the different teams on each challenge as well as we depict some conclusions we can extract from the results.

### 5.1 Polyp detection

**Table 5** presents global polyp detection results. We can observe that all teams achieve similar scores on the global metric results (mAP) even using different base architectures. Methods based on Visual Transformers (AURORA and CVC) appear to perform slightly better and in a more stable way in terms of performance ranking when we consider different thresholds about the minimum IoU value allowed for a correct detection.

**Table 5:** Results obtained on CVC-VideoClinicDB test set.

Team	mAP	*m*AP_50_	*m*AP_75_	F1	mIoU
CVC	**0.360**	0.654	**0.351**	0.809	0.592
AURORA	0.353	0.642	0.348	0.877	0.628
BYDLab	0.329	0.640	0.304	**0.902**	0.561
AI-JMU	0.351	**0.663**	0.326	0.833	**0.708**

Best results for each metric are highlighted in bold.

**Figure 2:**
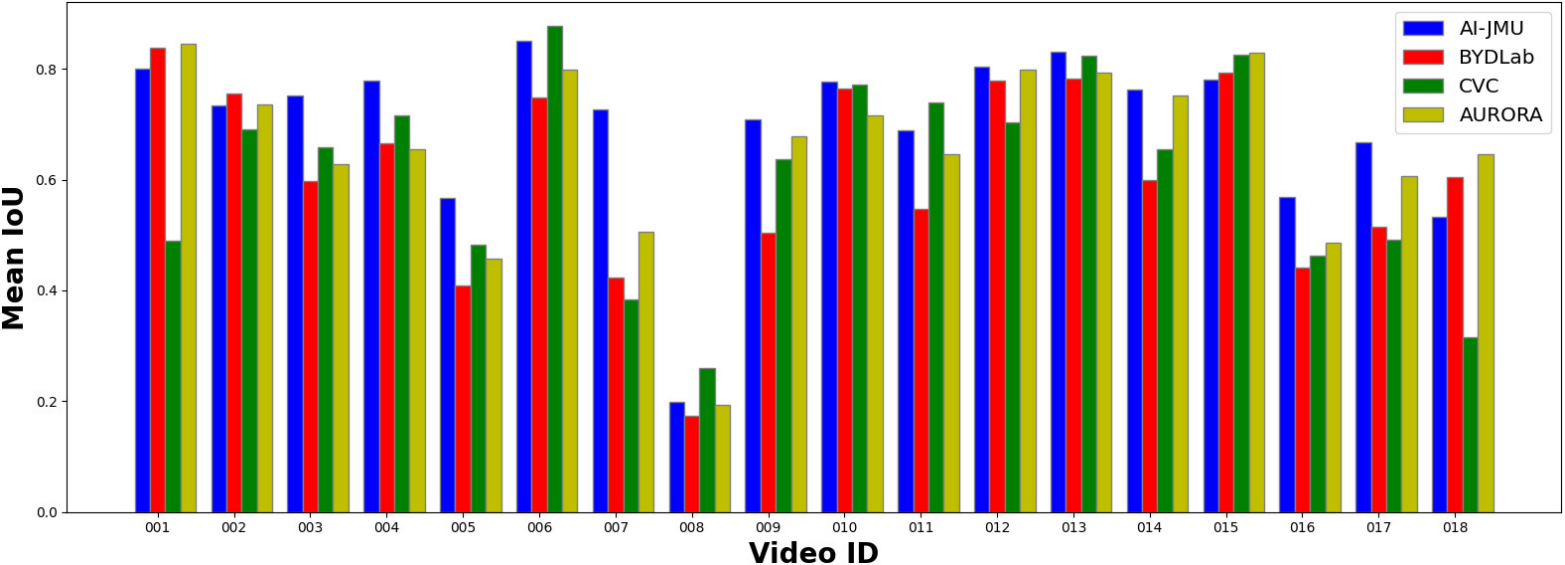
Mean IoU per video and team.

**Figure 2** presents the mean Intersection over Union (IoU) for each team across the different test sequences. First, we can observe that the majority of the teams consistently achieve an IoU score above 0.40 for all video sequences.

Nevertheless, there is a performance drop in sequences 5, 8, and 16.

We further analyzed these sequences to find some of the possible reasons why this happens. Sequence 5 contains a lot of frames with fecal content that obstruct polyp’s view and therefore makes it more difficult to detect.

The polyp in sequence 8 is very close to a fold, which makes it difficult to isolate the lesion from the surrounding region. Finally, sequence 16 contains a lot of frames where the scene is overexposed, making it difficult to properly differentiate any endoluminal structure.

**Figure 3:**
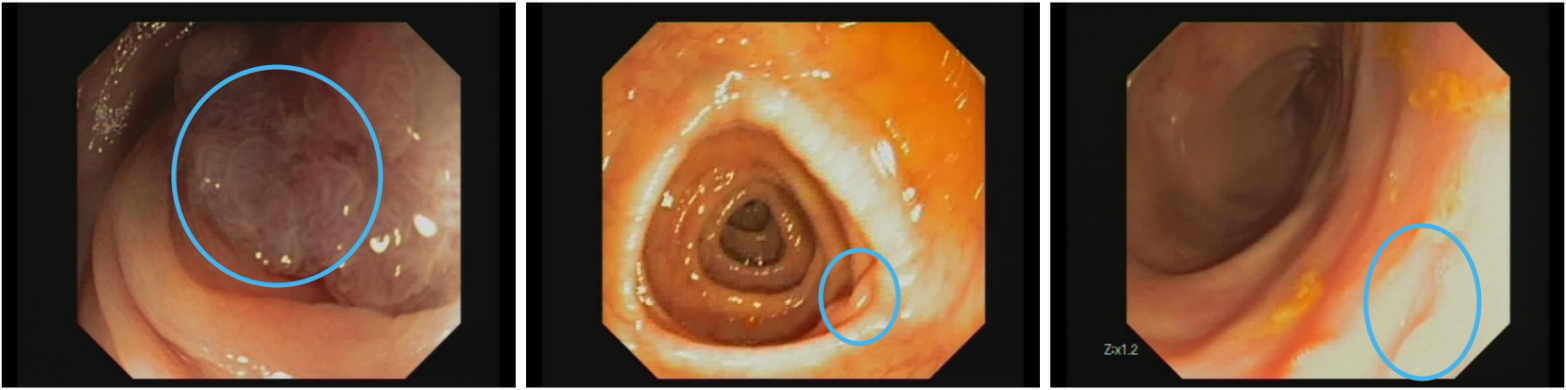
Examples of frames where polyp detection methods fail. Left: fecal substance covering polyp surface. Middle: austral fold hiding polyp. Right: overexposed region on the image. Images are extracted from CVC-VideoClinicDB dataset (36).

**Figure 3** displays selected frames highlighting these challenging conditions. It is clear from these sequences that poor visibility is the primary obstacle to reliable polyp detection.

**Figure 4:**
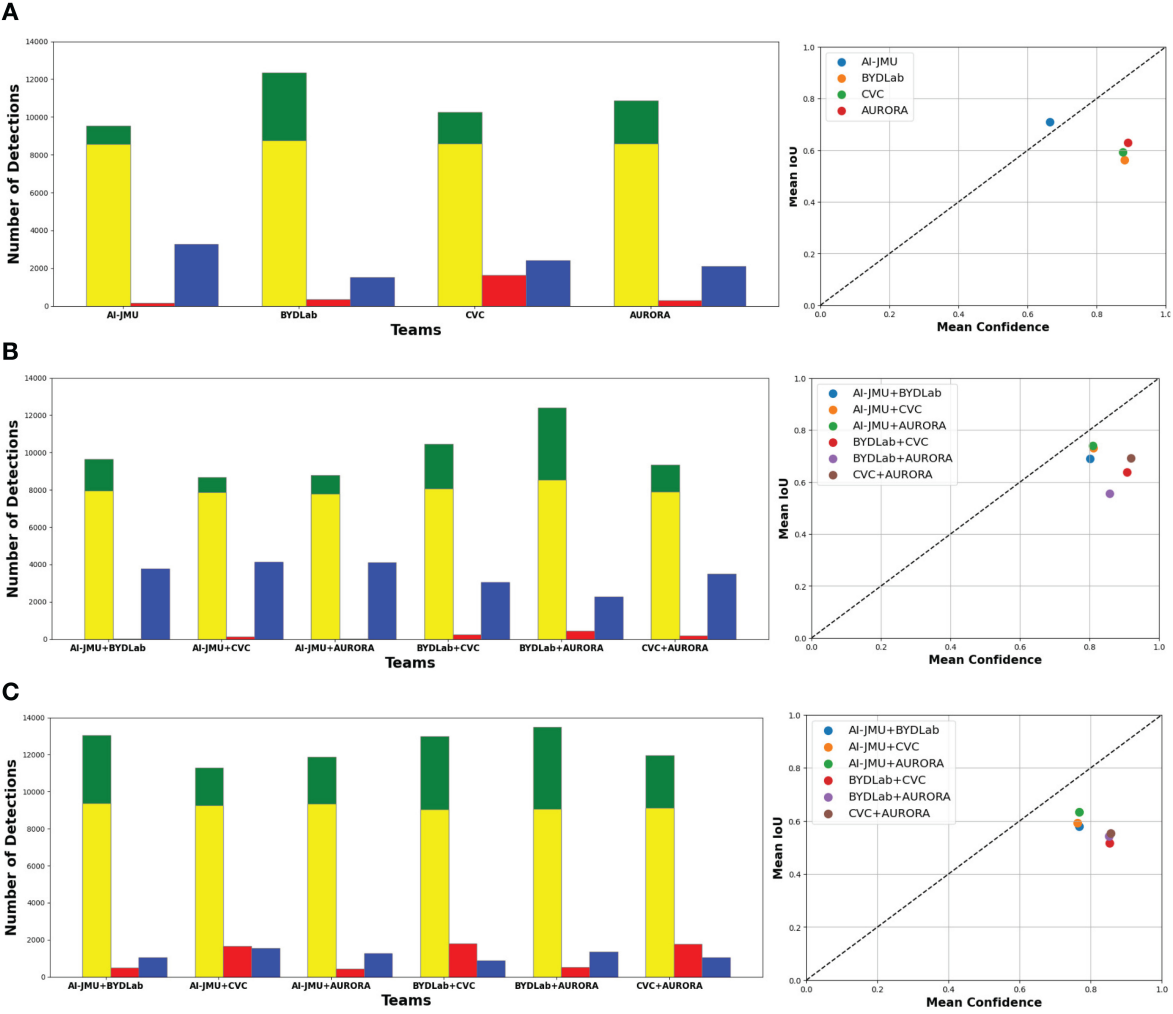
**(A)** Individual team comparison. **(B)** Combined Team Comparison using ‘AND’ method. **(C)** Combined team comparison using ‘OR’ method. Left: Correct Detections (green), Correct detections with +0.5 IoU (yellow), Missed Detections (blue), Extra Detections (red). Right: Model Calibration Plot.

To better understand the differences between the detection strategies of the teams, **Figure 4** presents additional data on how each team’s approach performs. A noteworthy observation is that AI-JMU achieves a higher rate of strong detections, which are those surpassing the 0.5 IoU threshold, suggesting a more precise detection capability, though it does not necessarily achieve the greatest overall number of correct detections.

**Figure 5:**
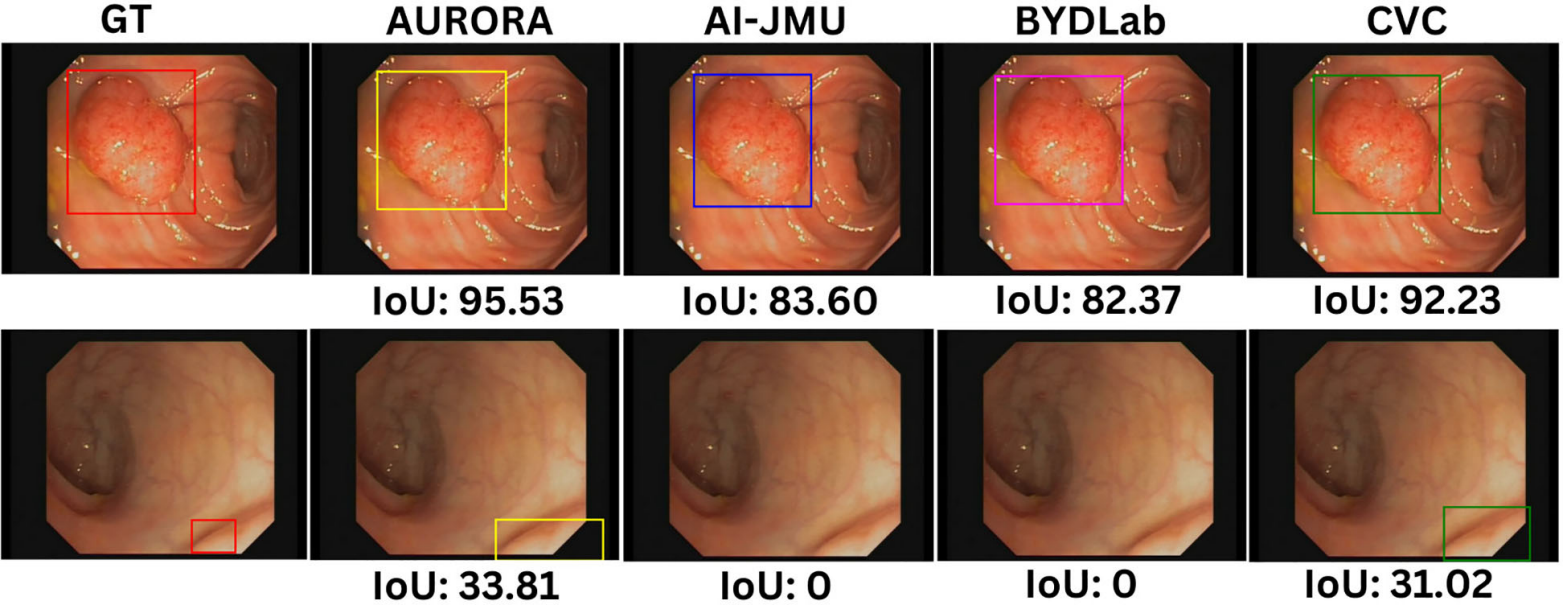
Examples of detection bounding boxes for each team with its IoU scores. Images are extracted from CVC-VideoClinicDB dataset (36).

**Figure 5** shows different detection results, two per team. In the first one (up) all teams detect the polyp but get different IoU scores when compared with Ground Truth and in the second one only some of teams do detect the polyp.

Beyond individual assessments, we merged different pairs of methods to investigate potential performance enhancements. We adopted ‘AND’ and ‘OR’ strategies for combining detections. If both teams provide a detection for a frame, both ‘AND’ and ‘OR’ approaches return a single bounding box by averaging the coordinates of the vertices of the original bounding boxes. However, if only one of the teams provides a detection, ‘OR’ strategy outputs the detected bounding box, while ‘AND’ returns none.

The ‘AND’ strategy results in a more conservative outcome, where only unanimous detections are considered, greatly reducing false positives but increasing the likelihood of missed detections. The ‘OR’ strategy, conversely, results in a higher detection rate but at the cost of increased false positives. In both approaches, if both teams detected a polyp in a given frame, we used the mean of both team’s bounding boxes as the final bounding box.

Additionally, the calibration graph in the right column of **Figure 4** visualizes each method’s performance, mapping confidence against detection precision (mean IoU). Ideally, methods should aspire to reside above the diagonal line (AI-JMU in this comparison study), indicating a harmonious balance between detection confidence and accuracy.

Methods lying below the diagonal line are likely to be overoptimistic about their predictions.

Finally, **Tables 6**, **7** present Reaction Time results. First we can observe that the majority of teams have a very low mean Reaction Time, in all cases smaller than a second, which can be interpreted as an almost instantaneous detection.

**Table 6:** Reaction Times (in frames) by Team and Video ID.

Team	01	02	03	04	05	06	07	08	09	10	11	12	13	14	15	16	17	18
AI-JMU	0	0	0	0	40	0	0	39	0	0	0	3	0	0	0	12	7	33
BYDLab	0	0	0	0	0	0	10	8	0	0	0	0	0	0	0	1	6	12
CVC	0	0	0	0	107	0	156	33	0	0	0	2	0	0	0	2	8	28
AURORA	0	0	0	0	0	0	8	8	0	0	0	0	0	0	0	0	6	12

**Table 7:** Global reaction time results: mean and standard deviation (in frames).

Team	Mean RT	Std RT
AI-JMU	7.44	13.77
BYDLab	2.06	3.87
CVC	18.67	41.81
AURORA	1.89	3.68

If we look at the results obtained for each video, we can also observe that the majority of the teams consistently detect the polyp as soon as it becomes visible however, certain videos, notably numbers 5, 7, 8, and 18, prove to be more challenging. Within this context, the BYDLab team emerges as the top performer, while CVC exhibits the smallest number of instantaneous detections.

### 5.2 Polyp segmentation

**Table 8** shows results from all the participating teams on SD and HD challenge. We can observe that the best methods offer the best performance in both SD and HD.

**Table 8:** Dice score and mIoU of each team on SD and HD segmentation test sets.

Team	SD		HD	
	DICE	IoU	DICE	IoU
CVC	0.750	0.659	0.817	0.727
AURORA	0.855	**0.785**	0.920	0.727
UPF	**0.859**	0.784	**0.929**	**0.876**
HK-UST	0.582	0.502	0.865	0.799
UoN	0.586	0.482	-	-

Best results for each metric are highlighted in bold.

**Figure 6:**
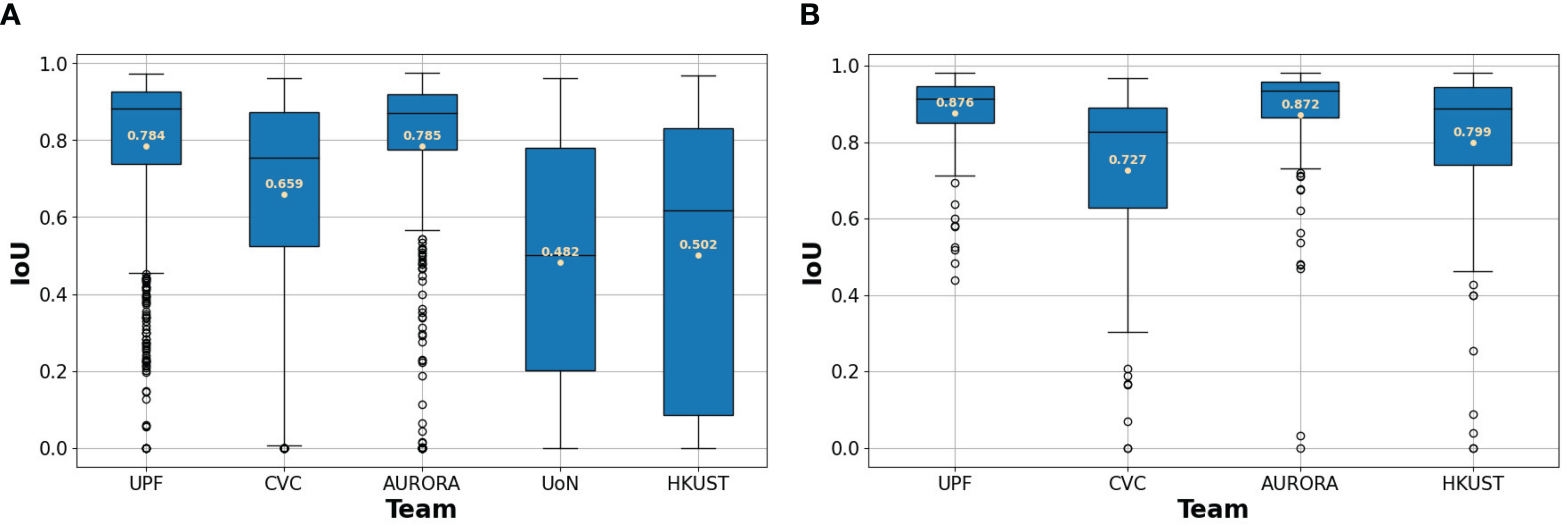
**(A)** Box plots for SD dataset. **(B)** Box plots for HD dataset. Box plots for each team’s IoU between their predicted segmentations and the ground truth. The mean IoU for each team is written in beige.

To better understand differences between methodologies, we present box plots in **Figure 6**. By looking at them, we could infer that polyp segmentation in HD images is easier than in SD images, as all the teams get substantially better metrics on this test set.

**Figure 7:**
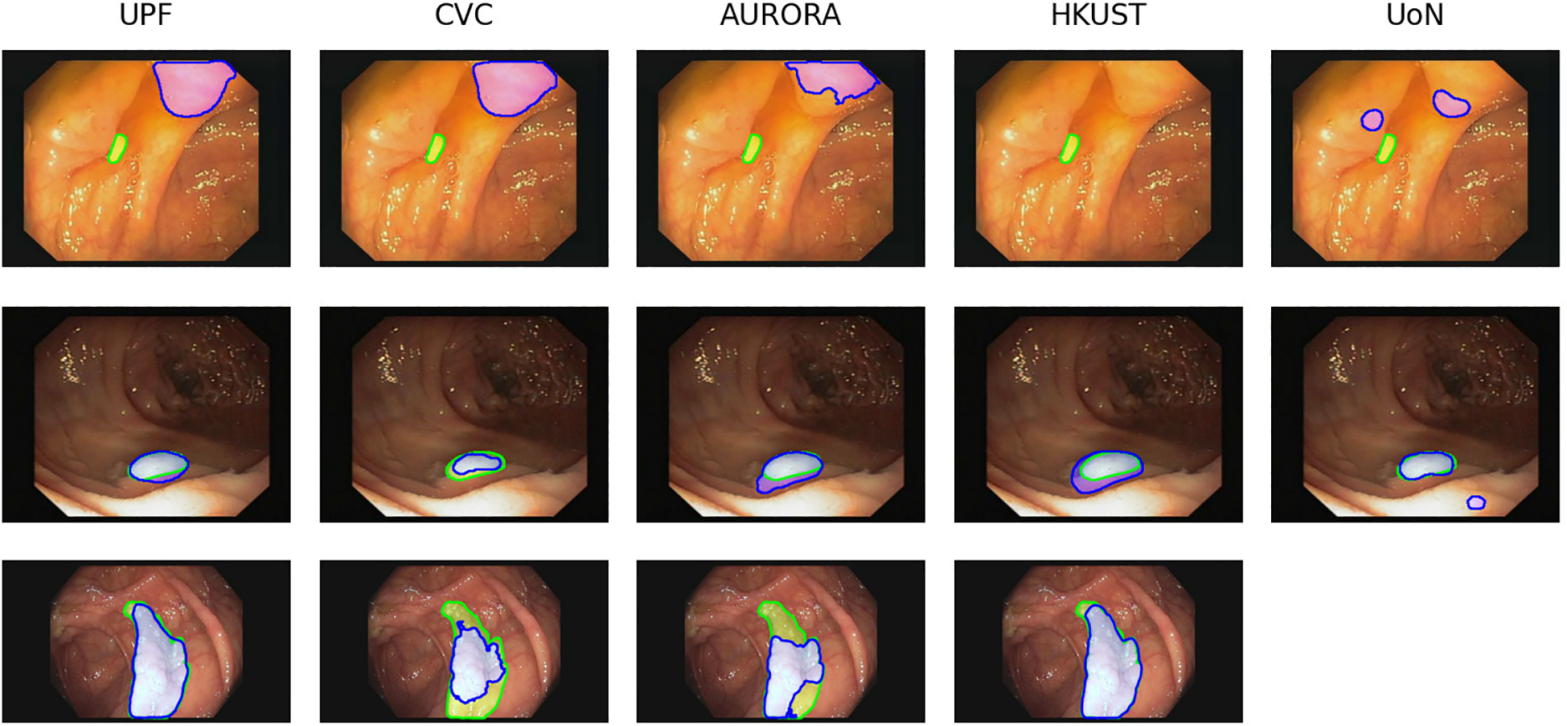
Examples of images from SD dataset (first and second row) and HD dataset (third row) with the corresponding segmentation predictions from each team. The ground truth masks are represented in green, whilst the predictions are shown in blue. Images are extracted from CVC- ColonDB (31) and CVC-ClinicDB (30) datasets.

In order to evaluate the performance of the methods, we computed the mean IoU between the predicted masks and the ground truth masks. We selected those frames with lower values to analyze the results. **Figure 7** shows three examples from the analyzed images. In the case of the first row, we can see that all the teams scored an IoU of zero between their predictions and the ground truth. The second row shows a sample where the polyp is easy to detect and all the teams’ predictions intersect with the ground truth mask. Note that some teams over-segment, such as AURORA and HK-UST, while other teams under-segment, as it is the case of CVC. The last row shows a sample where some of the predicted masks only cover the polyp partially.

The analysis of this particular example shows one of the problems that polyp detection and segmentation share: the similar appearance between polyps and another endoluminal structures, folds in this case, which results in false detections and incorrect segmentations.

### 5.3 Polyp classification

**Table 9** shows a summary of all the metrics derived from the analysis of the performance of the different methods.

**Table 9:** Confusion matrices and derived metrics from CVC-HDClassif test set.

Team	TP	FP	TN	FN	Prec	Rec	Spec	NPV	Acc	F1	F2	MCC
AURORA	126	15	57	27	89,36	82,35	79,17	67,86	81,33	85,71	83.66	0,59
Team AB	127	**12**	**60**	26	**91,37**	83,01	**83,33**	69,77	**83,11**	**86,99**	84.55	**0,64**
UPF	119	22	50	34	84,40	77,78	69,44	59,52	75,11	80,95	79.01	0,46
BYDLab	125	14	58	28	89,93	**81,70**	80,56	67,44	81,33	85,62	83.22	0,60
CVML	**134**	25	47	**19**	84,28	**87,58**	65,28	**71,21**	80,44	85,90	**86.90**	0,54
Best combinations	147	31	41	6	82,58	96,08	56,94	87,23	83,56	88,82	93,04	60,84

Best results for each metric are highlighted in bold.

We observe that Team AB and CVML are the ones that obtained a better overall performance. Team AB method is the one that obtains the best overall result but if we analyze the results from clinical usability perspective (where it is also important to classify correctly the non-adenomatous) CVML method should also be taken in consideration.

The last row in the table gathers the metrics of the best combinations of teams. Team combinations have been performed using logical ‘OR’ between predictions: if one of the teams classifies a sample as positive, the combined prediction for that sample is set to positive.

Considering this, there are three different combinations that achieve the same performance, namely: a) CVML + LSJLab + Team AB, b) CVML + Team AB + AURORA and c) CVML + LSJLab + Team AB + AURORA.

**Figure 8:**
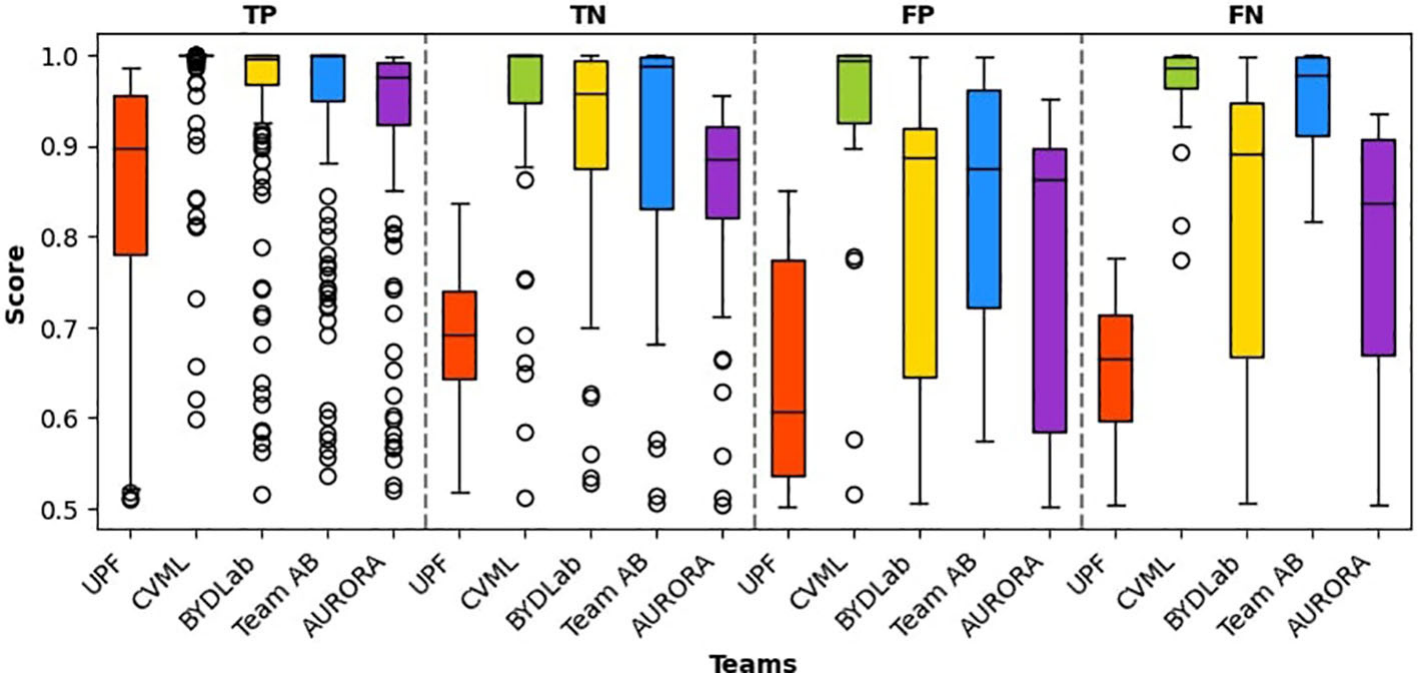
Box plots for each team’s confidence in polyp classification. It is worth noting that all confidence scores surpass 0.5, given the binary nature of the classification task.

We represent in **Figure 8** box plots showing the confidence that each of the teams provided in their predictions broken down by the actual outcome of the classification. All the teams achieved best mean confidence for adenoma classification (True Positives). We can also observe a higher standard deviation regarding confidence in both false positives and false negatives. Finally, we can observe how all the teams present higher confidence in correct classifications (TP and TN) than in incorrect ones (FP and FN).

Similar to the analysis done for polyp detection, we analyzed the results to see if there are some miss-classifications where all the teams fail. Most of the cases correspond to images that have features that are normally present on the opposite class.

**Figure 9:**
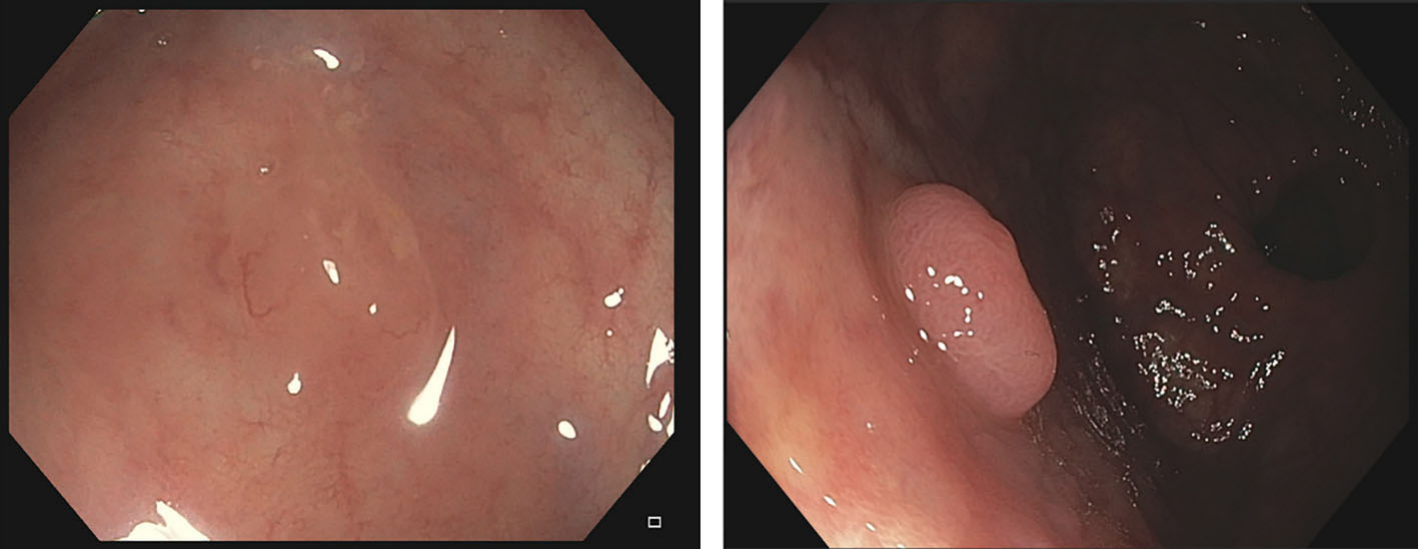
Images from CVC-HDClassif database that all the methods miss-classify. Left should be classified as adenomatous; Right should be classified as non-adenomatous.

We can see in **Figure 9** two examples where this happens: in the image on the left we can see an example of an adenomatous polyp that is plain and with low granulation, which are features commonly related from non-adenomatous class; in the image on the right we can observe a non-adenomatous polyp that has a lot of tubularities and a relatively high contrast with the surroundings.

**Figure 10:**
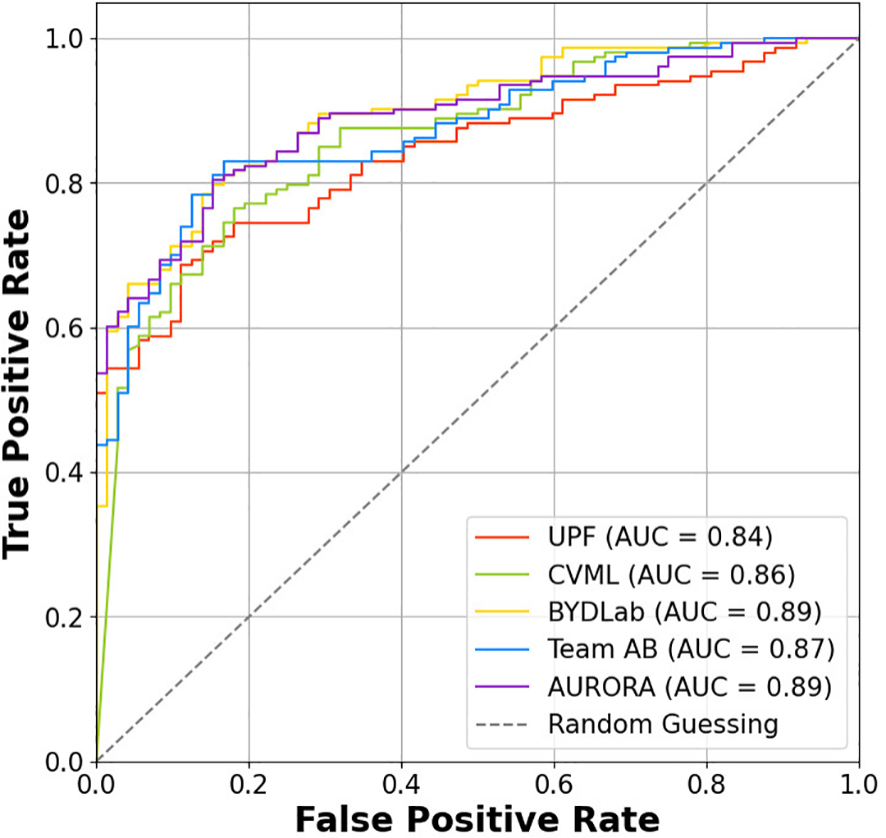
ROC curve for each team.

We provide in **Figure 10** a ROC curve to represent the performance of the different methods. We can observe that both AURORA and BYDLab offer the highest AUC score, though there are no big differences among the teams.

## 6 Discussion

We have introduced in this paper a complete validation framework for polyp detection, segmentation and classification in colonoscopy images. Our aim was not only to detail the full framework, but also to evaluate whether existing methodologies are ready for practical use in the exploration room. In this section we will discuss the results of each of the different polyp characterization tasks along with diving into the general limitations present in both the methodologies and in the whole research field.

### 6.1 Polyp detection

Polyp detection has matured significantly, as evidenced by the narrowing performance gaps among existing methods. Nevertheless, this analysis has been performed over a relatively big dataset thoroughly reviewed by clinicians and that has already several years of use in the community. Despite this, the dataset has limitations, such as a lack of diversity in polyp appearances, which affects the robustness of the trained models. We will discuss later how this should be approached.

The architectures for polyp detection presented in this paper can be divided into two groups: those using Transformers (e.g., CVC and AURORA) and those employing more traditional approaches like Faster R-CNN (BYDLab) and Yolov5 (AI-JMU). Experimental results showed similar overall performance across methodologies. However, a Wilcoxon rank-sum test (as shown in **Table 10**) indicated significant differences in performance among individual videos, particularly between methods using similar architectures, like CVC and AURORA.

**Table 10:** p-value test from the mAP per video distributions.

	CVC	AURORA	BYDLab	AI-JMU
CVC	–	0,601	0,824	0,715
AURORA	0,601	–	0,924	0,911
BYDLab	0,824	0,924	–	0,837
AI-JMU	0,715	0,911	0,837	–

All detection methods in the challenge met the real-time constraint of processing a frame within 40 ms at a 25 fps frame rate, making them viable for real-time deployment in the exploration room, having most of them room to apply post-processing or even tracking methods to improve their results.

### 6.2 Polyp segmentation

After analyzing the results presented in the previous section, we can observe that there is a notable performance gap in polyp segmentation between standard definition (SD) and high definition (HD) images. Higher resolution and better texture information in HD images led to improve the quality of polyp masks across all the methods. Encoder-decoder networks (e.g., DPN from UPF) and transformer-based networks (e.g., Aurora) showed no significant performance differences.

In **Table 11** we show the results of Wilcoxon rank-sum test on the Intersection over Union (IoU) per image indicated similar distributions for AURORA and UPF in both SD and HD images (p-values: 0.08 and 0.12 respectively), with some variations in other methods. This correlates with the results, since those methods perform well on both well and produce similar predictions in terms of IoU.

**Table 11:** Results from a p-test comparing IoU values obtained from each of the images in SD and HD datasets for each of the methods.

	SD					HD				
Team	UoN	CVC	AURORA	HK-UST	UPF	UoN	CVC	AURORA	HK-UST	UPF
UoN	–	*<* 0.05	*<* 0.05	0.34	*<* 0.05	–	*<* 0.05	*<* 0.05	*<* 0.05	*<* 0.05
CVC	*<* 0.05	–	*<* 0.05	*<* 0.05	*<* 0.05	*<* 0.05	–	*<* 0.05	*<* 0.05	*<* 0.05
AURORA	*<* 0.05	*<* 0.05	–	*<* 0.05	0.08	*<* 0.05	*<* 0.05	–	*<* 0.05	0.12
HK-UST	0.34	*<* 0.05	*<* 0.05	–	*<* 0.05	*<* 0.05	*<* 0.05	*<* 0.05	–	*<* 0.05
UPF	*<* 0.05	*<* 0.05	0.08	*<* 0.05	–	*<* 0.05	*<* 0.05	0.12	*<* 0.05	–

With respect to the potential deployment of the presented approaches in a clinical setting, some segmentation methods require post-processing operations to achieve the final segmentation mask, which increases overall processing time and may prevent their real-time application.

### 6.3 Polyp classification

Polyp classification remains an immature field, with existing methods failing to meet the minimum performance required by clinicians. According to ASGE guidelines, a negative predictive value for non-adenomas smaller than 5 mm in the rectum-sigma region should exceed 90% (67).

In **Table 12** we selected the subset of polyps in test-set that are located in both rectum and sigma regions, and since the best method obtains a NPV of 58.82 (CVML), we concluded that none of the participants achieved the ASGE requirements to be effectively used as a CADx system in the exploration room.

**Table 12:** Confusion matrices and derived metrics from test set on rectum-sigma diminutive polyps.

Team	TP	FP	TN	FN	Prec	Rec	Spec	NPV	Acc	F1	F2	MCC
AURORA	13	9	10	18	59.09	41.93	52.63	35.71	46.00	49.05	44.52	-0.05
Team AB	16	9	10	15	64.00	51.61	52.63	40.00	52.00	57.14	53.69	-0.08
UPF	17	12	7	14	58.62	54.83	36.84	33.33	48.00	56.66	55.55	0.04
BYDLab	14	**8**	**11**	17	63.63	45.16	**57.89**	39.28	50.00	52.83	47.94	0.02
CVML	**24**	9	10	**7**	**72.62**	**77.41**	52.63	**58.82**	**68.00**	**75.00**	**76.43**	**0.30**

Best results for each metric are highlighted in bold.

Confusion matrices from **Table 9** and performance metrics reveal imbalanced class distributions, making accuracy an insufficient metric. From this we can see two approaches: methods like CVML that focus on detecting nonmalignant lesions, while others, like Team AB, aim for a balanced detection that performs better in terms of accuracy but at the cost of obtaining lower NPV.

Analyzing the approaches applied to tackle the polyp classification challenge, we have identified three different groups of approaches:

1. Classical image classification (analysis of the image as a whole): Those type of methods do not rely on segmentation data and, because of this, they are usually less expensive on the training phase.
2. Use of the same architecture for detection and classification: In this case, these methods learn jointly to localize and categorize polyps in a given frame. This can produce a problem when dealing with small datasets, in which we could potentially have not enough different samples for a method to generalize properly.
3. Classification from the output of the segmentation stage: In those cases, the class prediction is done by obtaining a segmentation map for each one of the classes or complementing the network by using segmentation as auxiliary task. Those methods share some of the weakness mentioned on the previous approach; if segmentation is not done properly and with representative data, the classification would also fail to achieve good performance.

Both winner (Team AB) and second best teams (CVML) could be linked to the first group of method as both of them rely on Efficient-Net as their base architecture. Although, it is clear that there is room for improvement in this task, particularly for the case of the minority class (non-adenomatous). This can be clearly seen by the generally low specificity scores obtained by the majority of approaches.

### 6.4 Limitations of the study

A significant challenge when developing robust systems, independently of the task, is to tackle the variability and scarcity of data. The lack of variability produces models that are prone to overfit towards those shapes and representation that are more represented, making models less generalizable. In the case of classification, the problem is even more critical: available data is poor in both variability and representativity of the different classes, resulting in datasets have the small number of samples with non-adenomatous lesions. This is due to current clinical protocols, where clinicians typically avoid removing these non-malignant lesions to reduce perforation risk and unnecessary pathological assessments.

Another limitation that arises from this validation study is that most of the methods are trained and tested over still images and, inherently, the approaches lack temporal information which could be beneficial to improve the models. For instance, we could solve some of the errors associated by having a polyp shot where the actual histology cannot be properly determined by the quality of the image or the presence of other endoluminal scene elements, as it can be seen in **Figure 3**.

Those methods usually learn discriminative features from the texture of the lesion and their surroundings, but if the selected frame has the lesion occluded or saturated the model will not perform as intended since the uncertainty will be high. As mentioned in section 5.1 sequences that obtain poor mAP results are due to the sequence present most of their frames with occluded shots.

## 7 Conclusions and future work

We have presented in this paper a complete validation framework for the analysis of polyp characterization methods in white light colonoscopy. We present a comparative analysis of different methodologies in the context of GIANA 21 challenge. After a deep analysis of the performance provided by the different methods, we can observe that some of the tasks appear to be more mature than others, particularly polyp detection and segmentation which have already appeared in other iterations of the challenge.

With respect to polyp detection, we observe a similar performance by all the participants regardless the base architecture. All the teams that we have compared in this study are able to detect all the different polyps in the dataset and, in the vast majority of the cases, the lesion is detected as soon as it appears in the video.

Even though, we observe that there are statistically significant differences when videos are analyzed individually and that there are some specific polyps where all the teams struggle, which makes it clear that more data is needed to build generalizable methods ready to be efficiently used in the exploration room.

Regarding polyp segmentation, we observe that there are logical differences associated to image resolution and degree of texture information but that the gap between the good level performance offered by the difference methods is small, showing that the field is already mature and that the task, for this particular data, is close to be solved.

This is not what happens with polyp classification, where results obtained by the different approaches show that there is still work to be done. Particularly, there is a need to balance the performance achieved for both adenomatous and non-adenomatous polyps, even considering that data is not balanced, as it happens in real life.

With respect to the future work to be carried out, regarding the validation framework we would like to extend the video database for polyp detection, potentially including HD videos and, with the addition of lesion histology, aiming at polyp classification using the same data. Even taking this into account and also related to polyp classification, efforts should be made to improve the balance between adenomatous and non-adenomatous lesions.

Besides, more histological classes such as serrated sessile adenomas should be included in order to reflect the evolution of clinical needs with respect to *in-vivo* histology prediction. Finally, virtual chromoendoscopy data (NBI for instance) could be acquired and labelled as its use has been proved to help clinicians to more accurately determine lesion histology in actual procedures.

## Data Availability

The original contributions presented in the study are included in the article/supplementary material. Further inquiries can be directed to the corresponding author.
