# Correction: A complete benchmark for polyp detection, segmentation and classification in colonoscopy images

**DOI:** 10.3389/fonc.2026.1798432

**Published:** 2026-02-20

**Authors:** Yael Tudela, Mireia Majó, Neil de la Fuente, Adrian Galdran, Adrian Krenzer, Frank Puppe, Amine Yamlahi, Thuy Nuong Tran, Bogdan J. Matuszewski, Kerr Fitzgerald, Cheng Bian, Junwen Pan, Shijle Liu, Gloria Fernández-Esparrach, Aymeric Histace, Jorge Bernal

**Affiliations:** 1 Computer Vision Center and Computer Science Department, Universitat Autònoma de Cerdanyola del Valles, Barcelona, Spain; 2 Department of Information and Communication Technologies, SymBioSys Research Group, BCNMedTech, Barcelona, Spain; 3 Artificial Intelligence and Knowledge Systems, Institute for Computer Science, Julius-Maximilians University of Würzburg, Würzburg, Germany; 4 Division of Intelligent Medical Systems, German Cancer Research Center (DKFZ), Heidelberg, Germany; 5 Computer Vision and Machine Learning (CVML) Research Group, University of Central Lancashir (UCLan), Preston, United Kingdom; 6 Hebei University of Technology, Baoding, China; 7 Tianjin University, Tianjin, China; 8 Digestive Endoscopy Unit, Hospital Clínic, Barcelona, Spain; 9 ETIS UMR 8051, École Nationale Supérieure de l’Électronique et de ses Applications (ENSEA), Centre national de la recherche scientifique (CNRS), CY Paris Cergy University, Cergy, France

**Keywords:** computer-aided diagnosis, medical imaging, polyp classification, polyp detection, polyp segmentation

An incorrect number was provided. The correct **Funding** number is PID2020–120611RB-I00. The current **Funding** statement reads: "The author(s) declare financial support was received for the research, authorship, and/or publication of this article. This work was supported by the following Grant Numbers: PID2020-120611RB-I00 and RED2022-134964-T and funded by MCIN-AEI/10.13039/501100011033. AG was funded by a Marie Sk lodowska-Curie Global Fellowship (No 892297). AH and JB thanks the Institute of Advanced Studies from CY Paris Cergy University, Invited Prof. Position grant, through which the position was obtained in the context of “SmartVideocolonoscopy” project. AK and FP kindly thank the University Hospital of Würzburg and the Interdisziplinäres Zentrum für Klinische Forschung (IZKF) for supporting the research. AY and TT are supported by a Twinning Grant of the German Cancer Research Center (DKFZ) and the Robert Bosch Center for Tumor Diseases (RBCT). BM was supported by the Science and Technology Facilities Council grant number ST/S005404/1 and Kerr Fitzgerald by UCLan PhD grant."

The original version of this article has been updated.

